# Genetic Association Analysis of Complex Diseases Incorporating Intermediate Phenotype Information

**DOI:** 10.1371/journal.pone.0046612

**Published:** 2012-10-19

**Authors:** Yafang Li, Jian Huang, Christopher I. Amos

**Affiliations:** 1 Department of Genetics, The University of Texas MD Anderson Cancer Center, Houston, Texas, United States of America; 2 Department of Statistics and Actuarial Science, The University of Iowa, Iowa City, Iowa, United States of America; 3 Department of Community and Family Medicine, Geisel College of Medicine, Dartmouth College, Hanover, New Hampshire, United States of America; National Taiwan University, Taiwan

## Abstract

Genetic researchers often collect disease related quantitative traits in addition to disease status because they are interested in understanding the pathophysiology of disease processes. In genome-wide association (GWA) studies, these quantitative phenotypes may be relevant to disease development and serve as intermediate phenotypes or they could be behavioral or other risk factors that predict disease risk. Statistical tests combining both disease status and quantitative risk factors should be more powerful than case-control studies, as the former incorporates more information about the disease. In this paper, we proposed a modified inverse-variance weighted meta-analysis method to combine disease status and quantitative intermediate phenotype information. The simulation results showed that when an intermediate phenotype was available, the inverse-variance weighted method had more power than did a case-control study of complex diseases, especially in identifying susceptibility loci having minor effects. We further applied this modified meta-analysis to a study of imputed lung cancer genotypes with smoking data in 1154 cases and 1137 matched controls. The most significant SNPs came from the *CHRNA3-CHRNA5-CHRNB4* region on chromosome 15q24–25.1, which has been replicated in many other studies. Our results confirm that this *CHRNA* region is associated with both lung cancer development and smoking behavior. We also detected three significant SNPs—rs1800469, rs1982072, and rs2241714—in the promoter region of the *TGFB1* gene on chromosome 19 (*p* = 1.46×10^−5^, 1.18×10^−5^, and 6.57×10^−6^, respectively). The SNP rs1800469 is reported to be associated with chronic obstructive pulmonary disease and lung cancer in cigarette smokers. The present study is the first GWA study to replicate this result. Signals in the 3q26 region were also identified in the meta-analysis. We demonstrate the intermediate phenotype can potentially enhance the power of complex disease association analysis and the modified meta-analysis method is robust to incorporate intermediate phenotype or other quantitative risk factor in the analysis.

## Introduction

Genome-wide association (GWA) studies have identified hundreds of common genetic variants associated with complex diseases and provided valuable insight into their genetic architecture. However, most of these variants confer relatively low risk effects and explain only a small proportion of the heritability of most complex diseases. For most of these diseases, less than 10% of the genetic variance is explained by the identified common variants, leaving the bulk of heritability unexplained [1]. One important reason for the unexplained heritability is that most of the genetic variants that have been identified have small odds ratios (around 1.1 for the heterozygous genotypes and 1.5–1.6) for the homozygous genotypes; latent variants likely have even less of a disease effect [2]. Researchers estimated that hundreds of genetic variants are involved in the development of complex diseases but that together they would explain only about 20% of genetic variance [3]. Investigators have used imputation of genetic loci from the Hapmap and other referent populations to boost the power of case-control association studies for complex diseases [4]–[5]. However, traditional case-control studies still have limited power to detect genetic variants with low risk effect so new statistical analysis methods are needed in the study.

Disease status is often the ultimate result of influences from multiple genotypes and environmental factors. Many “intermediate” phenotypes reflect the pathway leading to disease development. An intermediate phenotype may reflect more directly the effects from causal genes than disease status and be less genetically complex and more strongly associated with susceptibility loci. Analysis of intermediate phenotypes has the potential to capture the underlying heritable trait variation that may be missed in case-control studies, thus increasing the statistical power in genetic association studies [6]–[7]. Studying intermediate phenotypes would also provide insight into the complicated etiologic disease pathways. Behavioral and other quantitative measures of increased risk for disease may also help to improve the power of studies to detect associations of genetic factors with disease risk if these behavioral or other risk factors involve the same genetic factors in their etiology as the disease.

Intermediate phenotypes, also known as endophenotypes, were first used in psychiatric disorders studies as they were easier to measure and less complicated than disease status [6]. Endophenotypes have been successfully applied in unraveling the complex etiology of mental disease. For example, neurological soft signs have been used as an endophenotype in analysis of Schizophrenia. In 2012, Greenwood et al. [8] found 94 candidate genes associated with Schizophrenia-relate endophenotypes. Simons et al. [9] identified VMAT2 as a candidate gene for psychotic disorder and neurocognition using measurement of cognitive functioning as the intermediate phenotype. Researchers also have adapted endophenotype for use in other complex disease studies. For example, high mammographic density is one of the strongest known risk factors for breast cancer and is an intermediate phenotype that can help elucidate the genetic factors that contribute to development of breast cancer [10]. In 2011, researchers identified the gene *ZNF365* on chromosome 10 as being associated with both breast cancer and mammographic density [11]. That same year, researchers used neuropathology and cognitive function proximate to death as the intermediate phenotypes for Alzheimer disease and identified two genes—*ZNF224* and *PCK1*—involved in the development of Alzheimer disease [12]. In these two studies, the researchers performed linear regression analysis of the quantitative intermediate phenotype with the marker genotype as the covariates. Their findings suggested successful use of intermediate phenotypes in genetic association analysis of complex diseases.

Meta-analysis is a powerful method in GWA studies, as it can combine information from independent populations, thus increasing the sample size and overcoming the lack of power in most common disease studies [13]–[16]. The combined information from multiple populations is either disease status or quantitative trait, not both of them. The most widely used meta-analysis techniques are Fisher's combined probability test [17] and inverse-variance weighting [18]. When intermediate phenotypes and disease status are both available in a study, a meta-analysis method combining disease status and intermediate phenotypes should be more powerful than either a case-control study or linear regression analysis of quantitative traits alone, as meta-analysis incorporates more information from the patients. In the present study, we demonstrated that meta-analysis can be used to examine a combination of the disease status and intermediate phenotype information from a single population in a complex disease study and a modified inverse-variance weighted method was proposed for the analysis. Simulation was conducted to evaluate the performance of Fisher's combined probability test, the modified inverse-variance weighted method, and the traditional case-control method. The results showed that inverse-variance weighting was the best of the three methods. We then applied the meta-analysis to a study of imputed lung cancer genotypes with smoking data. The results validated previous findings regarding the *CHRNA3-CHRNA5-CHRNB4* region on chromosome 15q24–25.1 [19]–[23] and the promoter region of the *TGFB1* gene on chromosome 19 [24]–[25], which suggested the modified inverse-variance weighting was a reliable method to do the meta-analysis within a study. A new region—3q26.1—was also identified; no genes are located in this region, and deletion of the region has been reported to be associated with some cancers [26]–[27].

## Results

### Simulation study of the novel method for combining results from disease and intermediate phenotype association studies

**Figure 1:**
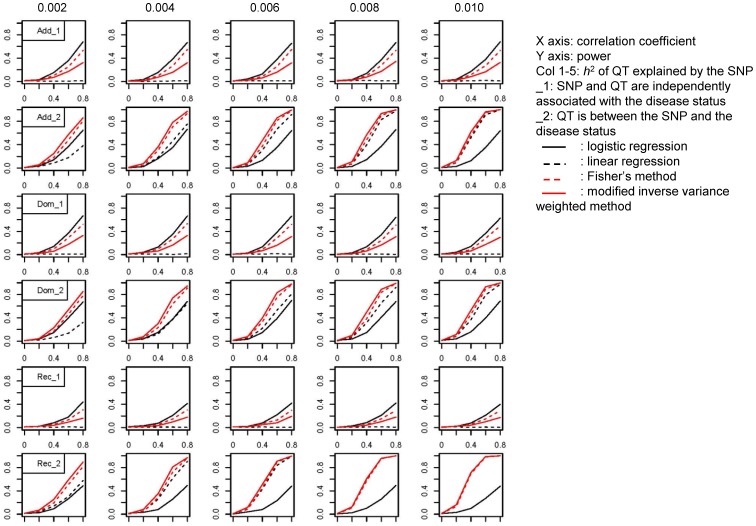
Power Plots for the Medium-Risk Model.

**Figure 2:**
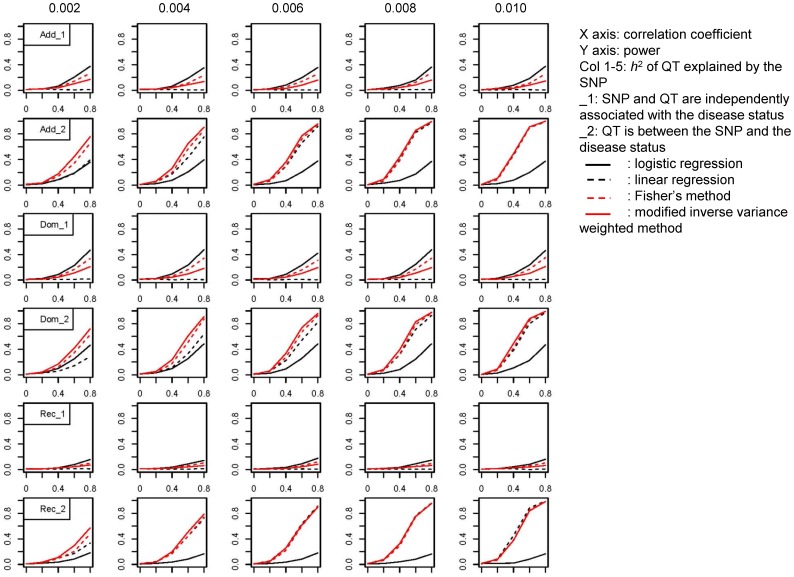
Power Plots for the Low-Risk Model.

Table 1 lists the parameters for the medium- and low-risk susceptibility loci in simulations. The results for the medium- and low-risk variants are shown in Figures 1 and 2. The x-axis in each graph denotes the correlation coefficient between the SNP marker and disease locus, which increased from 0 to 0.8. The y-axis in each graph denotes the power of each test. When the SNP marker was directly associated with the disease status but the disease-related quantitative trait was not associated with the SNP marker of interest, we obtained no useful information about the quantitative trait pertaining to the SNP marker studied (lines 1, 3, and 5 in Figures 1–2). Logistic regression analysis was the most powerful method to detect the association between the SNP marker and disease status followed by Fisher's combined probability test. The power of modified inverse-variance weighted method was only about half of that of logistic regression. When the quantitative trait was an intermediate phenotype between the SNP marker and disease status, linear regression analysis of the quantitative trait provided valuable information for the association analysis. The power of the tests increased as the correlation coefficient between the SNP marker and disease locus increased (x-axis). Also, as the heritability of the quantitative trait explained by the SNP increased from 0.002 to 0.010 (columns 1–5 in Figures 1–2), the power of the linear regression analysis increased, as did the power of the meta-analysis methods, because they rely on the information from linear regression analysis. The modified inverse-variance weighted method was more powerful than Fisher's combined probability test in the meta-analysis (lines 2, 4, and 6 in Figures 1–2). Using the recessive model, logistic regression analysis had little power, and the linear regression analysis had the predominant effect in the meta-analysis. The performance of Fisher's combined probability test and the modified inverse-variance weighted method were almost equal to that of the linear regression analysis.

**Table 1:** Parameters for Medium- and Low-Risk Models in simulation.

	Genetic Model	β_1_	γ_0_	γ_1_	OD_hetero_	OD_homo_
**Medium**	Add_1_ a	0	−3.36	log1.20	1.20	1.44
	Add_2_ b	1	−3.1	log1.15	1.20	1.43
	Dom_1_	0	−3.36	log1.14	1.30	1.30
	Dom_2_	1	−3.16	log1.07	1.31	1.31
	Rec_1_	0	−3.13	log1.14	1.00	1.30
	Rec_2_	1	−3.04	log1.07	1.00	1.31
**Low**	Add_1_	0	−3.16	log1.10	1.10	1.21
	Add_2_	1	−3.05	log1.05	1.10	1.22
	Dom_1_	0	−3.18	log1.08	1.17	1.17
	Dom_2_	1	−3.07	log1.04	1.17	1.17
	Rec_1_	0	−3.05	log1.08	1.00	1.17
	Rec_2_	1	−3.00	log1.04	1.00	1.17

OD_hetero_: odds ratio for heterozygous genotypes; OD_homo_: odds ratio for homozygous genotypes; Add: additive; Dom: dominant; Rec: recessive.

a Disease model 1 in Figure 1–2.

b Disease model 3 in Figure 1–2.

The type I error rate in this simulation was set at 0.01. To obtain an accurate estimation of the type I error rate, we carried out 10,000 simulations for each set of conditions under the null hypothesis of no association between the SNP marker and disease locus. We did not observe an inflated type I error rate in this simulation for any of the methods (Table S1 and S2).

### Application of the modified inverse-variance weighted meta-analysis method to imputed lung cancer genotypes with smoking data

**Figure 3:**
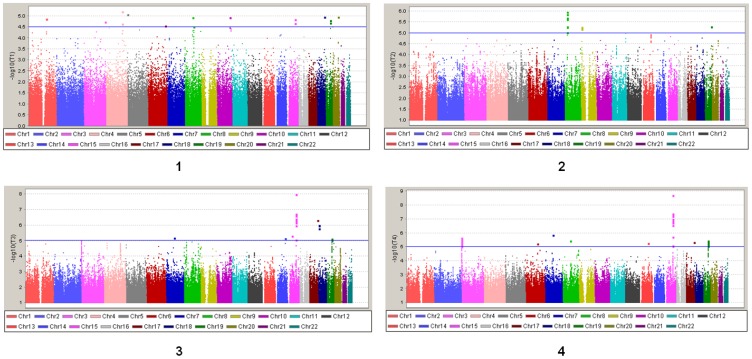
Manhattan Plot of GWA Studies of Lung Cancer and CPD Data. 1–4: case-control method (λ = 1.018), linear regression analysis with adjustment for disease status (λ = 1.010), Fisher's combined probability test (λ = 1.013), and the modified inverse-variance weighted method (λ = 1.011). −log_10_(p)>4.5 was used as the cutoff in plot 1 to match with the previous GWA study published in 2008 (Nat Genet, 40.5: 616–622). −log_10_(p)>5 was used as the cutoff in plot 2–4 to reduce false discovery rate.

**Figure 4:**
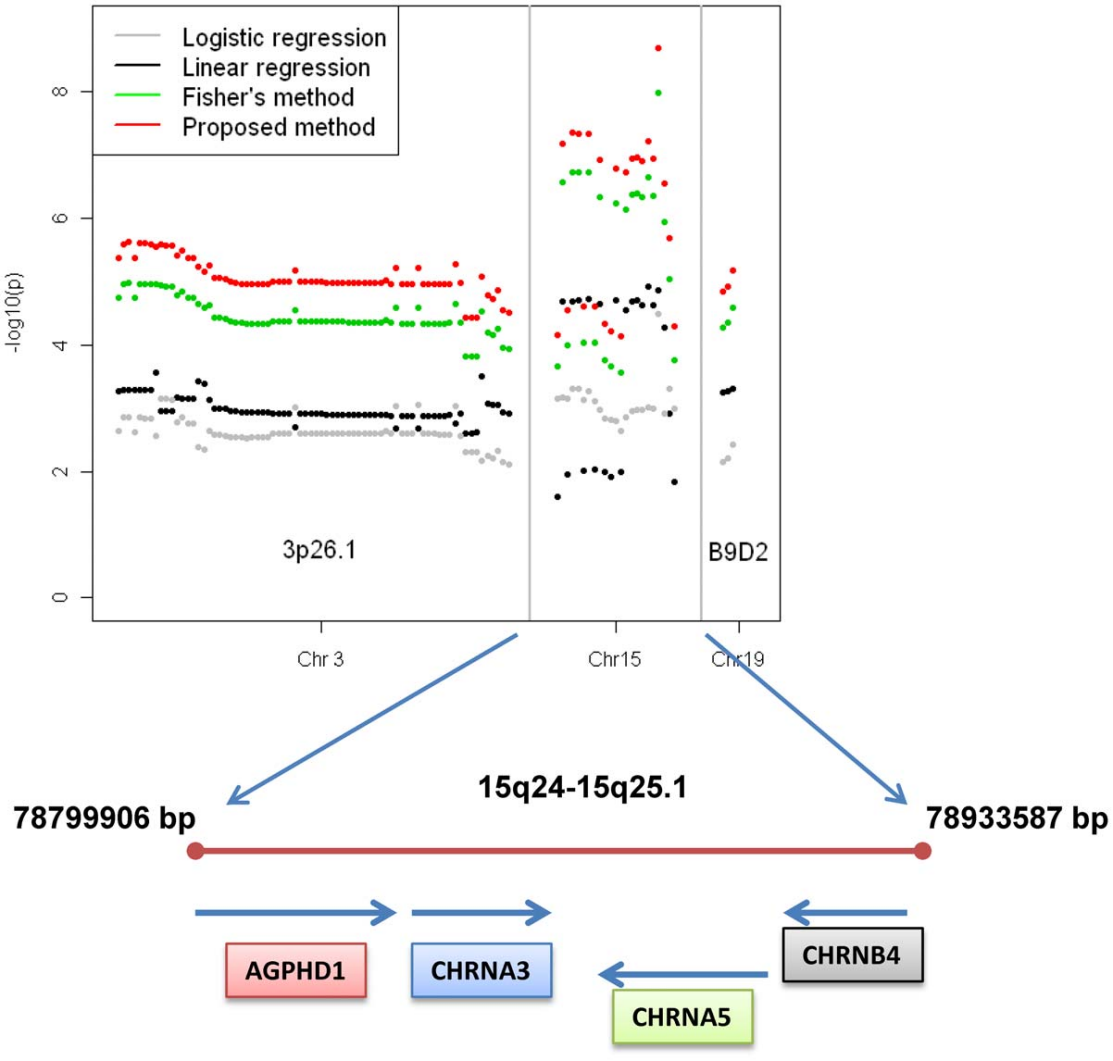
−Log_10_(P) Plot of Significant SNPs on Chromosomes 3, 15, and 19 in Meta-analysis of imputed lung cancer genotypes with smoking data.

The −log_10_(p)s for logistic regression analysis of disease status, linear regression analysis of cigarettes per day (CPD) with adjustment for disease status, Fisher's combined probability test, and our modified inverse-variance weighted method are plotted in Figure 3. The SNPs with −log_10_(p)s greater than 5 are highlighted as the SNPs potentially associated with lung cancer. Although the SNPs do not meet the commonly accepted criterion of −log_10_(p)>8 because of our limited sample size, they are still very promising signals that can be further validated. The inflation factors λ in the tests were ranged from 1.01–1.02, indicating no spurious association caused by population stratification in the analyses. Consecutive significant SNPs in a chromosomal region are listed in Table S3, and we identified three significant regions in our meta-analysis. The most significant region was *AGPHD1-CHRNA3-CHRNA5-CHRNB4* on chromosome 15q24–25.1 (Figure 4). When we used a traditional case-control method, no SNP in this region had a −log_10_(p) greater than 5 because of the limited sample size. In the meta-analysis, this region became very significant with the strongest signal at rs12914385 with a p-value 1.98×10^−9^. This result confirmed that the *CHRNA3-A5* region on 15q24–25.1 is associated with both lung cancer development and smoking behavior, which several other independent studies have already proven [19]–[23], and that CPD is an intermediate phenotype for lung cancer.

Another significant region is the *B9D2* gene, which encodes a protein that lies partially within the *TGFB1* promoter on chromosome 19 [28]. SNP rs1800469, rs1982072, and rs2241714 had a *p* value less than 0.001 in our case-control study and less than 0.01 in quantitative trait analysis. In the modified inverse-variance weighted meta-analysis, these three SNPs had a p-value of 1.46×10^−5^, 1.18×10^−5^, and 6.57×10^−6^, respectively. Previous authors reported that the SNPs rs1800469 and rs2241712 in the promoter of the *TGFB1* gene are associated with chronic obstructive pulmonary disease and lung cancer in cigarette smokers [24]–[25]. Our results supported the signal at rs1800469; rs2241712 was not present in our genotype data, but rs2241714 (about 350 bp away from rs2241712) was also significant (Table S3). The evidence from a GWA study supports that the *TGFB1* gene is associated with tobacco-induced lung cancer. The significant SNPs in the *TGFB1* promoter region may be related to abnormal *TGFB1* gene transcription levels in lung cancer patients. We also identified a large region on 3p26 (4.139–4.258 Mb) associated with both lung cancer development and smoking behavior, a total of 74 SNPs with a p-values around 1.0×10^−5^ were detected, and only two of them were from the genotyped SNPs, they were rs1444056 (4214953 bp) and rs1403124 (4188033 bp). No genes with known functions reside in this region although deletion of the region has been reported to be associated with some cancers [26]–[27].

**Figure 5:**
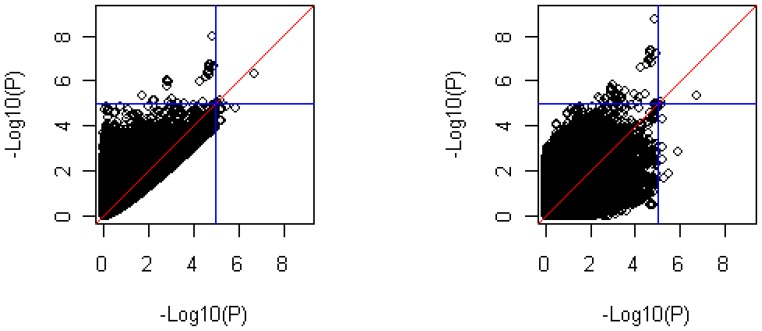
*P*-Value Comparisons between the tests. X-axis, −log_10_(p) from logistic regression analysis. Y-axis, −log_10_(p) from Fisher's combined probability test (left); −log_10_(p) from the modified inverse-variance weighted method (right).

For the significant SNPs identified in the three regions, the modified inverse-variance weighted method always produced a stronger signal than did Fisher's combined probability test. To further compare the performance of Fisher's combined probability test and the modified inversed-variance weighted method in association analysis, we plotted the −log_10_(p) in the case-control method versus Fisher's combined probability test, and the case-control method versus the inverse-variance weighted method (Figure 5). The plot on the left in the figure shows that Fisher's combined probability test tended to produce more significant signals for non-significant SNPs (−log_10_(p)<4) in the case-control study, which may have introduced a higher false-discovery rate than the inverse-variance weighted method in real data analysis. The reason for this finding is that Fisher's combined probability test is based on the *p* values from the logistic and linear regression tests, and so cannot tell the directions of the association effect from these two regression tests. Fisher's combined probability test can produce a significant result even when the effects in logistic and linear regression analyses are in opposite directions. This should not be true when the quantitative trait is an intermediate phenotype for the disease being studied. The modified inverse-variance weighted method does not have this problem because it is based on linear combination of the two effect sizes. Therefore, it is a better method than Fisher's test for a single- population meta-analysis.

## Discussion

Researchers have widely used meta-analysis in genetic association studies to combine information from different populations and increase sample sizes. However, it is rarely used to combine different types of data in a single population. Genetic researchers will often collect phenotypic information in addition to disease status to better understand the pathophysiology of disease development and to maximize study findings; in many of these instances, the information is on intermediate phenotypes. A meta-analysis method incorporating both the disease status and intermediate phenotype should be more powerful than a traditional case-control study method. In the present study, we examined a modified inverse-variance weighted meta-analytical method. Simulation studies showed that this method is more powerful than the traditional case-control method in association analysis of complex diseases, especially for identification of disease loci having very minor effects. Also, compared with Fisher's combined probability test, inverse-variance weighted meta-analysis is more robust as it has a bigger power and a lower type I error rate. We set the MAFs of the SNP marker and disease locus as equal in our simulation studies and we observed that the results of the tests were similar when the MAFs were set differently (results not shown). In addition, the intermediate phenotypes in both patients and controls were available in this study. This phenotype is sometimes only available in patients because either the quantitative trait is expressed in them only or the cost of measuring the quantitative trait in controls is too high. Our simulation study showed that the meta-analysis was still better than the case-control study method when the quantitative trait was only available for patients (results not shown).

We further applied meta-analysis to empirical data analysis. Smoking behavior, which can be quantified as smoking duration or smoking quantity, is the most important risk factor for lung cancer development. In 2008, several replicated studies showed that there was a strong association between the nicotinic acetylcholine receptor subunit cluster of genes (*CHRNA*) on chromosome 15q25.1 and lung cancer. But there was no conclusion on whether the association was direct or mediated via smoking behavior. Hung's group [23] observed an increased risk even in non-smokers, which implied at least some of the risk was not mediated via smoking. Thorgeirsson et al. [29] suggested that the association with lung cancer was mainly mediated through smoking behavior. In 2010, researchers using genome-wide approaches provided conclusive evidence for a strong association between *CHRNA* genes and smoking behavior [22]. There is reason to believe that *CHRNA* genes are associated with both smoking and lung cancer. Smoking behavior is an attribute associating with increased lung cancer risk. The method that we derive can be applied equally well to either intermediate phenotypes or to behavioral attributes that associate with increased risk for a disease. To address this comment, we revised our paper by inserting discussion about modeling either intermediate phenotypes or other quantitative risk factors into the model. The GWA study incorporating the quantitative trait of CPD with the imputed genotype data detected significant SNPs on chromosomes 3, 15, and 19. The signal in the *CHRNA3-CHRNA5-CHRNB4* region was much stronger in the meta-analysis than in the case-control study. The highest *p* value was 1.98×10^−9^, which was a very strong signal in our small sample size (1154 cases and 1146 controls). Many independent studies have replicated the finding of association of *CHRNA3-CHRNA5-CHRNB4* on 15q24 with lung cancer and smoking behavior. Our results further confirmed this finding. Also, it suggested that CPD is abehaviorally mediated risk factor for lung cancer or an intermediate phenotype that is involved in lung cancer risk. Whether or not the genetic effects of the nicotinic receptor variants on chromosome 15q25.1 directly contribute to lung cancer risk or only contribute through their effects on smoking behavior is a topic of ongoing debate and further study. Mediation analyses [30]–[31] have shown both direct and indirect (through smoking behavior) effects of the SNPs in this region on lung cancer risk. In contrast, a study of the SNP effects on cigarette per day use versus cotinine levels among smokers shows a much stronger effect on cotinine levels [32]–[33]. This finding suggests that reported cigarettes per day is inadequately capturing the actual exposure individuals experience to nicotine, but this observation still does not indicate yet the exact pattern of relationship of the genetic effects on smoking versus lung cancer risk [34].

The SNPs rs1800469 and rs2241712 in the promoter of the *TGFB1* gene on chromosome 19 were associated with chronic obstructive pulmonary disease and lung cancer in smokers in previous studies. These polymorphisms can only be detected in our study using meta-analysis. Thus, meta-analysis combining an intermediate phenotype and the disease status is a powerful tool for detecting genetic variants in complex disease association studies, especially when the effects of the susceptibility loci are minor. The significant SNPs detected in these verified regions demonstrate that our modified inverse-variance weighted meta-analysis is a reliable method for genetic association studies when an intermediate phenotype is available.

In the lung cancer study, the intermediate or behaviorally related phenotype, smoking quantity, has a positive relationship with disease status. This positive correlation may not always be true. For example, there is a negative relationship between brain size and Alzheimer's disease. In this case, the quantitative trait can be specified as the measurement of the overall brain shrinkage from the patient's normal brain size, which has a positive relationship with the disease. Researchers may use prior studies to assess correlations between the intermediate phenotype and the disease of interest to help determine how this information should be combined in the joint analysis.

In this study, the modified inverse-variance based test was applied when only one intermediate phenotype is available. Statistically, it can also be applied when multiple intermediate phenotypes are available in the data as this method is based on the combination of estimators from several regression tests with the modified inverse variance as the weights. However, consideration is needed on the complicated disease model when multiple intermediate phenotypes are existent. The disease model could include multiple disease pathways with each one having an intermediate phenotype in it, or one pathway with more than one intermediate phenotypes in it, or even a mixture of them. Further investigation is needed for the application of this method in a more complicated situation.

Whereas an intermediate phenotype is very useful in GWA studies, it also has potential to help researchers understand the intricate interactions among the disease associated genes and elucidate the complicated mechanism underlying the human diseases. The rapid development of microarray technology has made genome-wide gene expression profiles available to researchers. The gene expression levels are closely linked with both the genetic variants and disease status, providing a large number of intermediate phenotypes for complex diseases. Meta-analysis combining the disease status and gene expression data will be very powerful in identifying the functional genetic variants associated with complex diseases. This modified inverse-variance weighted meta-analytic approach is a promising tool in deciphering complex disease codes.

## Materials and Methods

### Simulation study

Given a disease locus A having two alleles A_1_ and A_2_ with allele frequencies q_1_ and q_2_ (q_1_+q_2_ = 1), an SNP marker M has two alleles M_1_ and M_2_ with allele frequencies m_1_ and m_2_ (m_1_+m_2_ = 1). The SNP marker and disease locus are closely linked so that they are in linkage disequilibrium, which can be quantified using the correlation coefficient (*r*). The pathway to a complex disease has an intermediate phenotype that can be measured as a quantitative trait (Y). If X denotes the genotype at the SNP marker, then the relationship between Y and X can be expressed using the linear equation Y = β_0_+β_1_X+ε, in which ε represents the error term following N(0,σ^2^_E_). The genotypes at X were coded as 0, 1, and 2 for an additive effect; 0, 1, and 1 for a dominant effect; 0, 0, and 1 for a recessive effect. The relationship between the disease status and SNP marker and quantitative trait can be expressed using the equation P(D|X,Y) = exp(γ_0_+γ_1_X+γ_2_Y)/(1+exp(γ_0_+γ_1_X+γ_2_Y)).The minor allele frequencies (MAFs) of the SNP marker (m_1_) and disease allele are both set at 0.3 for a common allele frequency. The values for β and γ parameters in the logistic equation are chosen to fix the disease incidence rate at 0.05 (Table 1). The value of σ^2^_E_ represents the residual effect in the regression analysis, which includes the effect of environmental factors and impact of other genetic loci. The heterozygous and homozygous odds ratios at the SNP marker range from 1.2 to 1.4 for a medium-risk model and 1.1 to 1.2 for a low-risk model.

The genetic variance of the quantitative trait explained by the SNP marker can be expressed as σ^2^_A_ = 2m_1_m_2_α^2^, σ^2^_D_ = (2m_1_m_2_ak)^2^, in which α = a[1+k(m_1_−m_2_)] [35]. In this equation, *a* and *k* represent additive and dominant effects of the SNP marker respectively. In this simulation, the heritability of the quantitative trait explained by the SNP marker ranges from 0.002 to 0.010, with a step size of 0.002; these numbers are based on the estimation of common variants effect-size distribution from recent GWA studies in complex diseases [3]. The type I error rate in the simulation study was set at 0.01. Additive, dominant, and recessive effects were simulated at the SNP marker, although the additive model is the common model assumed in quantitative trait association analyses. For the medium-risk variants, we simulated 2000 cases and 2000 controls for the additive and dominant models and 6000 cases and 6000 controls for the recessive model. For the low-risk variants, we simulated 4000 cases and 4000 controls for the additive and dominant models and 8000 cases and 8000 controls for the recessive model. The analysis program is coded in R which is available upon request, and we have posted the code to SourceForge (http://sourceforge.net/p/modifiedinverse/wiki/Home/).

### Disease models in simulations

**Figure 6:**
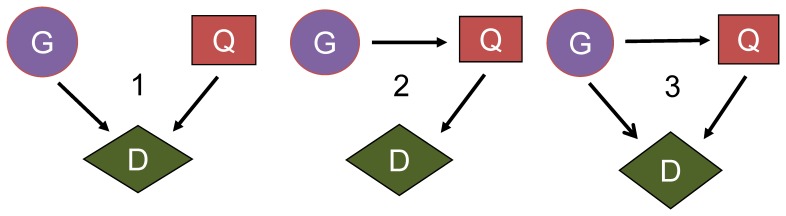
Three possible disease models for one disease locus with an intermediate phenotype. G, disease susceptibility locus; D, disease status; QT, quantitative trait (an intermediate phenotype).

Figure 6 lists the possible relationships among the disease susceptibility locus, disease status, and quantitative trait given this trait is an intermediate phenotype. In model 1, the quantitative trait and SNP marker are independently associated with the disease status, and the disease locus is not related to the quantitative trait. Models 2 and 3 are different from each other etiologically. Model 2 is a single pathway, whereas model 3 is a dual pathway between the disease susceptibility locus and disease status. Mediation analysis can be used to differentiate these two models [36], but it is impossible to separate models 2 and 3 when the disease status and quantitative trait are evaluated separately in the analysis. The results from disease models 1 and 3 were reported, with the correlation coefficient (*r*) between the SNP and disease susceptibility locus increasing from 0 to 0.8 [Figure 1–2]. When *r* equals 0, the SNP is not associated with the disease locus, which is the null hypothesis in the simulation.

### Analysis of simulated data

Two statistical tests were conducted for the analysis in step 1. Test 1 is logistic regression analysis of the disease status with the SNP marker genotype as the covariate, and test 2 is linear regression analysis of the quantitative trait with adjustment for the disease status. For the three proposed disease models under the assumption that the quantitative trait is an intermediate phenotype, no association between the SNP and disease status, either directly or indirectly, means there is no association between the SNP and quantitative trait and no association between the SNP and disease status. So test 1 and test 2 are independent under the null hypothesis. Step 2 is to combine the results from test 1 and 2 using meta-analysis methods. If p_1_ and p_2_ are the *p* values for test 1 and 2, Fisher's combined probability test can be used to combine the values to provide an overall *p* value using the formula χ^2^ = −2[ln(p_1_)+ln(p_2_)], which follows a chi-square distribution with four degrees of freedom [17].

Two Z-score statistics from tests 1 and 2 were obtained from score tests, which can be combined using inverse-variance weighting. Suppose the two Z-scores from tests 1 and 2 are Z_1_ and Z_2*_, both follow an approximately normal distribution as follows:
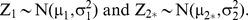
Under the null hypothesis, Z_1_ and Z_2*_ are independent (μ_1_ = μ_2*_ = 0).

The estimator of effect size of the SNP from linear regression analysis of the quantitative trait can be arbitrary depending on the subjective selection of the measurement unit and normalization procedure of the trait, which will affect the combination result from the inverse-variance weighted method. However, if the quantitative trait is an intermediate phenotype involved in the development of disease, the estimators of the coefficients for SNP marker from logistic and linear regression analysis and their standard errors should be close to a consistent unit, as these are two different tests for the same associations, i.e., the association between the SNP marker and disease status. Therefore, Z_2*_ can be scaled so that it has the same unit as Z_1_. In the present study, the standard error of Z_2*_ is scaled so that it is the same as that of Z_1_, which is the same as multiplying each quantitative trait by a constant *c*, where c = sqrt(σ^2^_1_/σ^2^_2*_). This produces the new Z-score statistic Z_2_ = Z_1_c∼N(μ_2_,σ^2^_1_). Let L = bZ_1_+(1−b)Z_2_, 0<b<1, when b = 1/2, the variance in *L* is at its smallest, specifically, 1/2V(Z_1_) (Text S1). This creates the new statistic *S*, which follows a normal distribution: S = (bZ_1_+(1−b)Z_2_)/sqrt(1/2V(Z_1_)∼N(μ_3_,σ^2^_3_). In this formula, μ_3_ = 0 under the null hypothesis.

### Lung cancer and smoking data with imputed marker data

The study examined 1154 ever-smokers with lung cancer and 1137 control ever-smokers. The patients and controls were frequency-matched by age and sex, and they were all of European origin. Their genotype data came from Illumina HumanHap300 v1.1 BeadChips, and the GWA study results were published in 2008. The genotypes were further imputed using the MACH (version 1.0.15) [37] with the HapMap 2 database (release 21), which contained 2,557,253 tagging SNPs. The statistical tests were conducted on imputed genotypes. Smoking cigarettes per day (CPD) was used as a quantitative trait in the analysis. We used the smoking data of CPD as the intermediate phenotype in our analysis. The box plot and histogram in Figure S1 show the distribution of the CPD data, and the Q-Q plot in Figure S1 shows the normality of the CPD data. We used a square root transformation to normalize the CPD data.

SNPs with a −log_10_(p) greater than 5 were regarded as promising significant SNPs with adjustment for multiple comparisons in the association analysis. The normally accepted −log_10_(p)>8 was not used because of our limited sample size.

## Supporting Information

Figure S1 **Top, descriptive plots of CPD before aquare root transformation Bottom, descriptive plots of CPD after square root transformation.**
(DOC)

Table S1 **Type I Error Rates for Medium-Risk Variants in simulation.** Add: additive; Dom: dominant; Rec: recessive. ^a^Disease locus and quantitative trait are independently associated with the disease. ^b^Quantitative trait is intermediate between the disease locus and disease status. ^c^Test 1, logistic regression; test 2, linear regression; test 3, Fisher's combined probability test; test 4, modified inverse-variance weighted method.
(DOC)

Table S2 **Type I Error Rates for Low-Risk Variants in simulation.** Add: additive; Dom: dominant; Rec: recessive. ^a^Disease locus and quantitative trait are independently associated with the disease. ^b^Quantitative trait is intermediate between the disease locus and disease status. ^c^Test 1, logistic regression; test 2, linear regression; test 3, Fisher's combined probability test; test 4, modified inverse-variance weighted method.
(DOC)

Table S3 **Significant SNPs on Chromosomes 3, 15, and 19 in the Association Analysis.** CHR: chromosome; NA: not available. Test 1, logistic regression; test 2, linear regression analysis of CPD with adjustment for disease status; test 3, Fisher's combined probability test; test 4, modified inversx10-variance weighted method.
(DOC)

Text S1 **Inverse variance weighted combination of Z1 and Z2 has global minimum variance value.**
(DOC)
