# Supplementary material for: Genetic Association Analysis of Complex Diseases Incorporating Intermediate Phenotype Information
Source: PLoS One. 2012 Oct 19;7(10):e46612. doi: 10.1371/journal.pone.0046612 (PMC3477105; doi:10.1371/journal.pone.0046612)

**Figure S1** **Top, descriptive plots of CPD before aquare root transformation. Bottom, descriptive plots of CPD after square root transformation.**


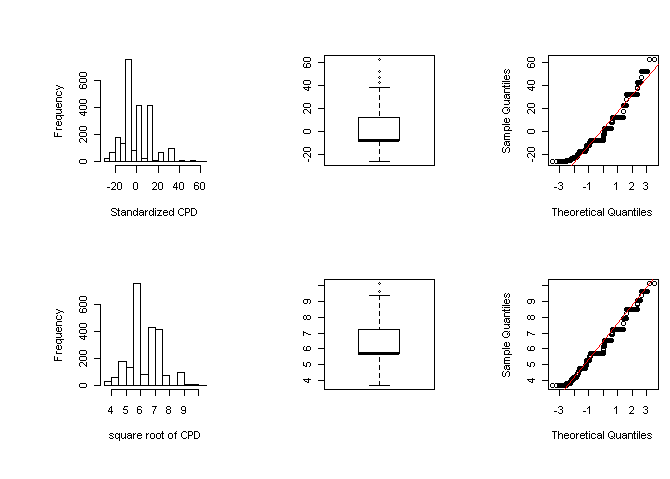

Supplement: Figure S1 — Top, descriptive plots of CPD before aquare root transformation Bottom, descriptive plots of CPD after square root transformation. (DOC) [file pone.0046612.s001.doc]
