# Supplementary material for: Genetic Association Analysis of Complex Diseases Incorporating Intermediate Phenotype Information
Source: PLoS One. 2012 Oct 19;7(10):e46612. doi: 10.1371/journal.pone.0046612 (PMC3477105; doi:10.1371/journal.pone.0046612)
Supplement: Table S1 — Type I Error Rates for Medium-Risk Variants in simulation. Add: additive; Dom: dominant; Rec: recessive. aDisease locus and quantitative trait are independently associated with the disease. bQuantitative trait is intermediate between the disease locus and disease status. cTest 1, logistic regression; test 2, linear regression; test 3, Fisher's combined probability test; test 4, modified inverse-variance weighted method. (DOC) [file pone.0046612.s002.doc]

**Table S1.** Type I Error Rates for Medium-Risk Variants in simulation

|  |  | |  | |  | | **Heritability^a^** | | |  | |  | |  | | **Heritability^b^** | | |  | |  |
| --- | --- | --- | --- | --- | --- | --- | --- | --- | --- | --- | --- | --- | --- | --- | --- | --- | --- | --- | --- | --- | --- |
| **Genetic Model** | | **Test^c^** | | **0.002** | | **0.004** | | **0.006** | **0.008** | | **0.01** | | **0.002** | | **0.004** | | **0.006** | **0.008** | | **0.01** | |
| **Add** | | 1 | | 0.0101 | | 0.013 | | 0.0098 | 0.0096 | | 0.0111 | | 0.0097 | | 0.009 | | 0.0093 | 0.0102 | | 0.0091 | |
|  | | 2 | | 0.0096 | | 0.0096 | | 0.0096 | 0.0097 | | 0.0085 | | 0.0109 | | 0.0103 | | 0.0099 | 0.0112 | | 0.0106 | |
|  | | 3 | | 0.0084 | | 0.0101 | | 0.0102 | 0.0083 | | 0.01 | | 0.0083 | | 0.0091 | | 0.0099 | 0.0093 | | 0.0098 | |
|  | | 4 | | 0.0093 | | 0.0118 | | 0.0102 | 0.0083 | | 0.0108 | | 0.0105 | | 0.0085 | | 0.0096 | 0.0107 | | 0.01 | |
| **Dom** | | 1 | | 0.0097 | | 0.0084 | | 0.0101 | 0.0113 | | 0.0101 | | 0.0078 | | 0.0105 | | 0.0087 | 0.0114 | | 0.0101 | |
|  | | 2 | | 0.0104 | | 0.0094 | | 0.0089 | 0.0099 | | 0.0104 | | 0.0084 | | 0.011 | | 0.0099 | 0.0107 | | 0.0111 | |
|  | | 3 | | 0.0099 | | 0.0097 | | 0.0091 | 0.009 | | 0.0104 | | 0.0099 | | 0.0098 | | 0.0105 | 0.0115 | | 0.0117 | |
|  | | 4 | | 0.0105 | | 0.0105 | | 0.0102 | 0.01 | | 0.0095 | | 0.0086 | | 0.0095 | | 0.0091 | 0.0119 | | 0.0116 | |
| **Rec** | | 1 | | 0.0011 | | 0.0105 | | 0.0102 | 0.0074 | | 0.0098 | | 0.0106 | | 0.0096 | | 0.0097 | 0.0111 | | 0.0111 | |
|  | | 2 | | 0.0103 | | 0.0082 | | 0.0098 | 0.0087 | | 0.011 | | 0.0095 | | 0.0111 | | 0.0106 | 0.0107 | | 0.0107 | |
|  | | 3 | | 0.0111 | | 0.01 | | 0.0108 | 0.0082 | | 0.0078 | | 0.0102 | | 0.0097 | | 0.0097 | 0.0107 | | 0.0107 | |
|  | | 4 | | 0.0101 | | 0.0088 | | 0.0092 | 0.0087 | | 0.0099 | | 0.0103 | | 0.0102 | | 0.0102 | 0.0102 | | 0.0102 | |

Add: additive; Dom: dominant; Rec: recessive.

^a^Disease locus and quantitative trait are independently associated with the disease.

^b^Quantitative trait is intermediate between the disease locus and disease status.

^c^Test 1, logistic regression; test 2, linear regression; test 3, Fisher's combined probability test; test 4, modified inverse-variance weighted method.
