# Supplementary material for: Genetic Association Analysis of Complex Diseases Incorporating Intermediate Phenotype Information
Source: PLoS One. 2012 Oct 19;7(10):e46612. doi: 10.1371/journal.pone.0046612 (PMC3477105; doi:10.1371/journal.pone.0046612)
Supplement: Table S2 — Type I Error Rates for Low-Risk Variants in simulation. Add: additive; Dom: dominant; Rec: recessive. aDisease locus and quantitative trait are independently associated with the disease. bQuantitative trait is intermediate between the disease locus and disease status. cTest 1, logistic regression; test 2, linear regression; test 3, Fisher's combined probability test; test 4, modified inverse-variance weighted method. (DOC) [file pone.0046612.s003.doc]

**Table S2.** Type I Error Rates for Low-Risk Variants in simulation

|  |  |  |  | **Heritability^a^** | |  |  |  | **Heritability^b^** | |  |
| --- | --- | --- | --- | --- | --- | --- | --- | --- | --- | --- | --- |
| **Genetic Model** | **Test^c^** | **0.002** | **0.004** | **0.006** | **0.008** | **0.01** | **0.002** | **0.004** | **0.006** | **0.008** | **0.01** |
| **Add** | 1 | 0.0105 | 0.0081 | 0.0098 | 0.0095 | 0.0101 | 0.0099 | 0.0101 | 0.0101 | 0.0105 | 0.0092 |
|  | 2 | 0.01 | 0.0112 | 0.0109 | 0.0099 | 0.01 | 0.0107 | 0.0094 | 0.0111 | 0.0095 | 0.01 |
|  | 3 | 0.0107 | 0.0087 | 0.0089 | 0.0109 | 0.0095 | 0.011 | 0.0103 | 0.0107 | 0.0087 | 0.0103 |
|  | 4 | 0.0107 | 0.0083 | 0.0094 | 0.0109 | 0.0092 | 0.0123 | 0.0108 | 0.0112 | 0.0105 | 0.0112 |
| **Dom** | 1 | 0.0095 | 0.0096 | 0.0105 | 0.0081 | 0.0092 | 0.0097 | 0.0104 | 0.0121 | 0.0101 | 0.0109 |
|  | 2 | 0.0092 | 0.0093 | 0.0103 | 0.0111 | 0.0096 | 0.0114 | 0.0122 | 0.0098 | 0.009 | 0.0099 |
|  | 3 | 0.0112 | 0.0085 | 0.0105 | 0.0084 | 0.009 | 0.0095 | 0.0117 | 0.0106 | 0.01 | 0.0103 |
|  | 4 | 0.0099 | 0.0087 | 0.0105 | 0.0096 | 0.0091 | 0.011 | 0.0118 | 0.0102 | 0.0098 | 0.0094 |
| **Rec** | 1 | 0.0103 | 0.0102 | 0.0078 | 0.0087 | 0.009 | 0.01 | 0.0102 | 0.0109 | 0.0101 | 0.0092 |
|  | 2 | 0.0101 | 0.0102 | 0.0103 | 0.009 | 0.01 | 0.0081 | 0.0103 | 0.0115 | 0.0112 | 0.0107 |
|  | 3 | 0.009 | 0.0112 | 0.0088 | 0.0103 | 0.0087 | 0.0111 | 0.01 | 0.0106 | 0.0095 | 0.0103 |
|  | 4 | 0.0103 | 0.0104 | 0.0077 | 0.0105 | 0.0091 | 0.009 | 0.0106 | 0.0113 | 0.0101 | 0.0096 |

Add: additive; Dom: dominant; Rec: recessive.

^a^Disease locus and quantitative trait are independently associated with the disease.

^b^Quantitative trait is intermediate between the disease locus and disease status.

^c^Test 1, logistic regression; test 2, linear regression; test 3, Fisher's combined probability test; test 4, modified inverse-variance weighted method.
