# Supplementary material for: Genetic Association Analysis of Complex Diseases Incorporating Intermediate Phenotype Information
Source: PLoS One. 2012 Oct 19;7(10):e46612. doi: 10.1371/journal.pone.0046612 (PMC3477105; doi:10.1371/journal.pone.0046612)
Supplement: Table S3 — Significant SNPs on Chromosomes 3, 15, and 19 in the Association Analysis. CHR: chromosome; NA: not available. Test 1, logistic regression; test 2, linear regression analysis of CPD with adjustment for disease status; test 3, Fisher's combined probability test; test 4, modified inversx10-variance weighted method. (DOC) [file pone.0046612.s004.doc]

**Table S3. Significant SNPs on Chromosomes 3, 15, and 19 in the Association Analysis**

|  |  |  |  | p | value |  |  |
| --- | --- | --- | --- | --- | --- | --- | --- |
| CHR | SNP | Position | Test 1 | Test 2 | Test 3 | Test 4 | Gene |
| 3 | rs795308 | 4139098 | 5.32X10-04 | 2.33X10-03 | 1.81X10-05 | 4.31X10-06 | NA |
| 3 | rs795307 | 4139274 | 5.19X10-04 | 1.41X10-03 | 1.11X10-05 | 2.55X10-06 | NA |
| 3 | rs795305 | 4139898 | 5.05X10-04 | 1.37X10-03 | 1.05X10-05 | 2.40X10-06 | NA |
| 3 | rs795304 | 4140062 | 5.13X10-04 | 2.41X10-03 | 1.81X10-05 | 4.33X10-06 | NA |
| 3 | rs795301 | 4143641 | 5.04X10-04 | 1.42X10-03 | 1.08X10-05 | 2.48X10-06 | NA |
| 3 | rs7614356 | 4148839 | 5.06X10-04 | 1.43X10-03 | 1.09X10-05 | 2.51X10-06 | NA |
| 3 | rs7611166 | 4154312 | 5.07X10-04 | 1.43X10-03 | 1.10X10-05 | 2.53X10-06 | NA |
| 3 | rs2587924 | 4155069 | 2.71X10-04 | 2.71X10-03 | 1.11X10-05 | 2.76X10-06 | NA |
| 3 | rs795294 | 4159299 | 1.09X10-03 | 6.98X10-04 | 1.15X10-05 | 2.59X10-06 | NA |
| 3 | rs1843303 | 4160124 | 1.11X10-03 | 7.19X10-04 | 1.21X10-05 | 2.71X10-06 | NA |
| 3 | rs2587916 | 4160379 | 1.12X10-03 | 7.24X10-04 | 1.22X10-05 | 2.75X10-06 | NA |
| 3 | rs2629249 | 4164455 | 6.79X10-04 | 1.68X10-03 | 1.67X10-05 | 3.87X10-06 | NA |
| 3 | rs2629251 | 4164548 | 6.99X10-04 | 1.39X10-03 | 1.44X10-05 | 3.30X10-06 | NA |
| 3 | rs2629256 | 4165422 | 7.02X10-04 | 1.75X10-03 | 1.80X10-05 | 4.17X10-06 | NA |
| 3 | rs2686702 | 4165452 | 7.06X10-04 | 1.77X10-03 | 1.83X10-05 | 4.25X10-06 | NA |
| 3 | rs973177 | 4166172 | 3.77X10-04 | 4.18X10-03 | 2.26X10-05 | 5.84X10-06 | NA |
| 3 | rs2587912 | 4170565 | 4.06X10-04 | 4.58X10-03 | 2.64X10-05 | 6.86X10-06 | NA |
| 3 | rs1444053 | 4171996 | 7.30X10-04 | 2.28X10-03 | 2.38X10-05 | 5.63X10-06 | NA |
| 3 | rs1444050 | 4175077 | 1.00X10-03 | 2.65X10-03 | 3.67X10-05 | 8.74X10-06 | NA |
| 3 | rs2262390 | 4177180 | 1.01X10-03 | 2.68X10-03 | 3.73X10-05 | 8.90X10-06 | NA |
| 3 | rs2600135 | 4184896 | 1.04X10-03 | 2.73X10-03 | 3.89X10-05 | 9.28X10-06 | NA |
| 3 | rs1838305 | 4185967 | 1.11X10-03 | 2.83X10-03 | 4.28X10-05 | 1.02X10-05 | NA |
| 3 | rs2120512 | 4186943 | 1.12X10-03 | 2.86X10-03 | 4.37X10-05 | 1.05X10-05 | NA |
| 3 | rs1829725 | 4187908 | 1.14X10-03 | 2.91X10-03 | 4.54X10-05 | 1.09X10-05 | NA |
| 3 | rs1403124 | 4188033 | 1.15X10-03 | 2.94X10-03 | 4.60X10-05 | 1.10X10-05 | NA |
| 3 | rs7431486 | 4191660 | 1.15X10-03 | 2.93X10-03 | 4.60X10-05 | 1.10X10-05 | NA |
| 3 | rs17488019 | 4192326 | 1.15X10-03 | 2.93X10-03 | 4.60X10-05 | 1.10X10-05 | NA |
| 3 | rs1444061 | 4197709 | 1.16X10-03 | 2.93X10-03 | 4.61X10-05 | 1.11X10-05 | NA |
| 3 | rs1485269 | 4197976 | 1.16X10-03 | 2.93X10-03 | 4.61X10-05 | 1.11X10-05 | NA |
| 3 | rs2600116 | 4199731 | 1.21X10-03 | 2.54X10-03 | 4.21X10-05 | 1.00X10-05 | NA |
| 3 | rs2600115 | 4200026 | 1.21X10-03 | 2.55X10-03 | 4.22X10-05 | 1.00X10-05 | NA |
| 3 | rs2587950 | 4200251 | 1.21X10-03 | 2.54X10-03 | 4.23X10-05 | 1.00X10-05 | NA |
| 3 | rs1444059 | 4203369 | 1.22X10-03 | 2.54X10-03 | 4.24X10-05 | 1.01X10-05 | NA |
| 3 | rs2322589 | 4204371 | 2.04X10-03 | 9.86X10-04 | 2.84X10-05 | 6.61X10-06 | NA |
| 3 | rs1444060 | 4205231 | 1.22X10-03 | 2.54X10-03 | 4.25X10-05 | 1.01X10-05 | NA |
| 3 | rs2248840 | 4205956 | 1.23X10-03 | 2.54X10-03 | 4.27X10-05 | 1.01X10-05 | NA |
| 3 | rs1385485 | 4206751 | 1.23X10-03 | 2.54X10-03 | 4.27X10-05 | 1.01X10-05 | NA |
| 3 | rs1385484 | 4206861 | 1.24X10-03 | 2.54X10-03 | 4.31X10-05 | 1.02X10-05 | NA |
| 3 | rs1994272 | 4207201 | 1.24X10-03 | 2.54X10-03 | 4.31X10-05 | 1.02X10-05 | NA |
| 3 | rs1994270 | 4207535 | 1.25X10-03 | 2.54X10-03 | 4.32X10-05 | 1.03X10-05 | NA |
| 3 | rs1485252 | 4209458 | 1.25X10-03 | 2.54X10-03 | 4.33X10-05 | 1.03X10-05 | NA |
| 3 | rs2629245 | 4211036 | 1.25X10-03 | 2.54X10-03 | 4.35X10-05 | 1.03X10-05 | NA |
| 3 | rs2600144 | 4211126 | 1.26X10-03 | 2.54X10-03 | 4.35X10-05 | 1.03X10-05 | NA |
| 3 | rs6442870 | 4211575 | 1.26X10-03 | 2.54X10-03 | 4.37X10-05 | 1.04X10-05 | NA |
| 3 | rs996402 | 4212188 | 1.28X10-03 | 2.54X10-03 | 4.45X10-05 | 1.06X10-05 | NA |
| 3 | rs2587931 | 4212523 | 1.29X10-03 | 2.54X10-03 | 4.46X10-05 | 1.06X10-05 | NA |
| 3 | rs2587930 | 4212598 | 1.29X10-03 | 2.54X10-03 | 4.47X10-05 | 1.06X10-05 | NA |
| 3 | rs1485250 | 4213057 | 1.29X10-03 | 2.55X10-03 | 4.49X10-05 | 1.06X10-05 | NA |
| 3 | rs1485249 | 4213075 | 1.29X10-03 | 2.55X10-03 | 4.49X10-05 | 1.07X10-05 | NA |
| 3 | rs1485248 | 4213123 | 1.30X10-03 | 2.55X10-03 | 4.50X10-05 | 1.07X10-05 | NA |
| 3 | rs1485247 | 4213196 | 1.30X10-03 | 2.32X10-03 | 4.12X10-05 | 9.72X10-06 | NA |
| 3 | rs2587929 | 4213613 | 1.31X10-03 | 2.55X10-03 | 4.54X10-05 | 1.08X10-05 | NA |
| 3 | rs2629247 | 4213709 | 2.06X10-03 | 9.08X10-04 | 2.65X10-05 | 6.17X10-06 | NA |
| 3 | rs2629248 | 4213859 | 1.32X10-03 | 2.55X10-03 | 4.57X10-05 | 1.08X10-05 | NA |
| 3 | rs2600134 | 4214099 | 1.32X10-03 | 2.56X10-03 | 4.60X10-05 | 1.09X10-05 | NA |
| 3 | rs2587928 | 4214325 | 1.33X10-03 | 2.56X10-03 | 4.61X10-05 | 1.09X10-05 | NA |
| 3 | rs2629250 | 4214394 | 2.07X10-03 | 8.98X10-04 | 2.64X10-05 | 6.14X10-06 | NA |
| 3 | rs1444058 | 4214847 | 1.33X10-03 | 2.56X10-03 | 4.64X10-05 | 1.10X10-05 | NA |
| 3 | rs1444057 | 4214927 | 1.34X10-03 | 2.56X10-03 | 4.65X10-05 | 1.10X10-05 | NA |
| 3 | rs1444056 | 4214953 | 1.34X10-03 | 2.56X10-03 | 4.67X10-05 | 1.11X10-05 | NA |
| 3 | rs7645164 | 4216649 | 1.33X10-03 | 2.57X10-03 | 4.65X10-05 | 1.10X10-05 | NA |
| 3 | rs1121798 | 4218602 | 1.31X10-03 | 2.59X10-03 | 4.61X10-05 | 1.10X10-05 | NA |
| 3 | rs2120508 | 4222809 | 1.26X10-03 | 2.65X10-03 | 4.54X10-05 | 1.08X10-05 | NA |
| 3 | rs1304143 | 4224222 | 1.73X10-03 | 9.16X10-04 | 2.28X10-05 | 5.25X10-06 | NA |
| 3 | rs9829633 | 4224946 | 1.22X10-03 | 2.69X10-03 | 4.47X10-05 | 1.07X10-05 | NA |
| 3 | rs1485254 | 4225696 | 2.48X10-03 | 4.94X10-03 | 1.51X10-04 | 3.74X10-05 | NA |
| 3 | rs1485256 | 4225871 | 2.46X10-03 | 4.95X10-03 | 1.50X10-04 | 3.72X10-05 | NA |
| 3 | rs1351948 | 4227083 | 2.43X10-03 | 4.96X10-03 | 1.49X10-04 | 3.69X10-05 | NA |
| 3 | rs313684 | 4237598 | 3.10X10-04 | 6.87X10-03 | 2.99X10-05 | 8.40X10-06 | NA |
| 3 | rs313696 | 4243751 | 8.31X10-04 | 5.74X10-03 | 6.32X10-05 | 1.63X10-05 | NA |
| 3 | rs1242809 | 4248055 | 8.67X10-04 | 6.29X10-03 | 7.16X10-05 | 1.86X10-05 | NA |
| 3 | rs1242810 | 4248489 | 8.81X10-04 | 4.73X10-03 | 5.58X10-05 | 1.40X10-05 | NA |
| 3 | rs1242815 | 4252366 | 1.18X10-03 | 7.23X10-03 | 1.08X10-04 | 2.82X10-05 | NA |
| 3 | rs923094 | 4258507 | 1.21X10-03 | 7.69X10-03 | 1.17X10-04 | 3.07X10-05 | NA |
| 15 | rs9788682 | 76589641 | 2.57X10-02 | 6.94X10-04 | 2.13X10-04 | 7.06X10-05 | AGPHD1 |
| 15 | rs9788721 | 76589924 | 2.06X10-05 | 6.68X10-04 | 2.63X10-07 | 6.48X10-08 | AGPHD1 |
| 15 | rs7164594 | 76590112 | 1.13X10-02 | 7.06X10-04 | 1.02X10-04 | 2.86X10-05 | AGPHD1 |
| 15 | rs8034191 | 76593078 | 2.02X10-05 | 4.81X10-04 | 1.88X10-07 | 4.45X10-08 | AGPHD1 |
| 15 | rs10519203 | 76601101 | 1.99X10-05 | 4.89X10-04 | 1.89X10-07 | 4.48X10-08 | AGPHD1 |
| 15 | rs7163730 | 76601736 | 9.78X10-03 | 7.24X10-04 | 9.10X10-05 | 2.50X10-05 | AGPHD1 |
| 15 | rs8031948 | 76603112 | 1.85X10-05 | 5.30X10-04 | 1.90X10-07 | 4.58X10-08 | AGPHD1 |
| 15 | rs4461039 | 76604502 | 9.29X10-03 | 7.67X10-04 | 9.16X10-05 | 2.49X10-05 | AGPHD1 |
| 15 | rs931794 | 76613235 | 2.29X10-05 | 1.07X10-03 | 4.53X10-07 | 1.18X10-07 | AGPHD1 |
| 15 | rs2036534 | 76614003 | 1.01X10-02 | 1.43X10-03 | 1.76X10-04 | 4.69X10-05 | AGPHD1 |
| 15 | rs3813570 | 76619887 | 1.23X10-02 | 1.51X10-03 | 2.21X10-04 | 6.05X10-05 | PSMA4 |
| 15 | rs2036527 | 76638670 | 1.98X10-05 | 1.58X10-03 | 5.71X10-07 | 1.61X10-07 | NA |
| 15 | rs684513 | 76645455 | 1.04X10-02 | 2.26X10-03 | 2.74X10-04 | 7.25X10-05 | CHRNA5 |
| 15 | rs17486278 | 76654537 | 2.85X10-05 | 1.38X10-03 | 7.11X10-07 | 1.90X10-07 | CHRNA5 |
| 15 | rs7180002 | 76661048 | 2.02X10-05 | 1.12X10-03 | 4.20X10-07 | 1.12X10-07 | CHRNA5 |
| 15 | rs951266 | 76665596 | 2.00X10-05 | 1.08X10-03 | 4.04X10-07 | 1.07X10-07 | CHRNA5 |
| 15 | rs16969968 | 76669980 | 2.33X10-05 | 1.08X10-03 | 4.67X10-07 | 1.22X10-07 | CHRNA5 |
| 15 | rs1051730 | 76681394 | 1.19X10-05 | 9.67X10-04 | 2.22X10-07 | 6.05X10-08 | CHRNA3 |
| 15 | rs1317286 | 76683184 | 2.40X10-05 | 9.93X10-04 | 4.42X10-07 | 1.14X10-07 | CHRNA3 |
| 15 | rs12914385 | 76685778 | 1.40X10-05 | 3.28X10-05 | 1.03X10-08 | 1.98X10-09 | CHRNA3 |
| 15 | rs17487223 | 76711042 | 5.24X10-05 | 1.23X10-03 | 1.13X10-06 | 2.82X10-07 | CHRNB4 |
| 15 | rs12441088 | 76715319 | 1.23X10-03 | 4.88X10-04 | 9.21X10-06 | 2.08X10-06 | CHRNB4 |
| 15 | rs11634351 | 76731773 | 1.46X10-02 | 1.00X10-03 | 1.77X10-04 | 5.07X10-05 | NA |
| 19 | rs1800469 | 4.66E+07 | 5.66X10-04 | 7.10X10-03 | 5.39X10-05 | 1.46X10-05 | B9D2 |
| 19 | rs1982072 | 4.66E+07 | 5.38X10-04 | 6.06X10-03 | 4.45X10-05 | 1.18X10-05 | B9D2 |
| 19 | rs2241714 | 46561232 | 4.84X10-04 | 3.80X10-03 | 2.61X10-05 | 6.57X10-06 | B9D2 |

**CHR: chromosome; NA: not available.**

**Test 1, logistic regression; test 2, linear regression analysis of CPD with adjustment for disease status; test 3, Fisher’s** combined probability test**; test 4,** modified inversx10-variance weighted **method.**
