# Supplementary material for: Genetic Association Analysis of Complex Diseases Incorporating Intermediate Phenotype Information
Source: PLoS One. 2012 Oct 19;7(10):e46612. doi: 10.1371/journal.pone.0046612 (PMC3477105; doi:10.1371/journal.pone.0046612)
Supplement: Text S1 — Inverse variance weighted combination of Z1 and Z2 has global minimum variance value. (DOC) [file pone.0046612.s005.doc]

**Text S1** If *Z*_1_ and *Z*_2_ are independent and follow normal distributions,

Z_1_~N[µ_1_,V(Z_1_)], Z_2_~N[µ_2_,V(Z_2_)].

Let

Z=bZ_1_+(1-b)Z_2_, 0<b<1,

V(Z)=b^2^V(Z_1_)+(1-b)^2^V(Z_2_).

V(Z) is a function of b, and the second derivative of V(Z) with respect to b is greater than 0, so V(Z) has a global minimum value.

f’(b)=ǝV(Z)/ǝb=2bV(Z_1_)-2(1-b)V(Z_2_)=2b[V(Z_1_)+V(Z_2_)]-2V(Z_2_)

f’’(b)=ǝ^2^V(Z)/ǝb^2^=2[V(Z_1_)+V(Z_2_)]>0

when f’(b)= 2b[V(Z_1_)+V(Z_2_)]-2V(Z_2_)=0,

b=V(Z_2_)/(V(Z_1_)+V(Z_2_))=1/2

When b = 1/2, V(Z) reaches its minimum, which is 1/2V(Z_1_).

V(Z)=V(Z_1_)V(Z_2_)/(V(Z_1_)+V(Z_2_))=1/2V(Z_1_)
